# Legal capacities required for prevention and control of noncommunicable diseases

**DOI:** 10.2471/BLT.18.213777

**Published:** 2018-11-20

**Authors:** Roger S Magnusson, Benn McGrady, Lawrence Gostin, David Patterson, Hala Abou Taleb

**Affiliations:** a Sydney Law School, University of Sydney, New South Wales, 2006, Australia.; b Prevention of Noncommunicable Diseases Department, World Health Organization, Geneva, Switzerland.; c WHO Collaborating Center for National & Global Health Law, Georgetown University, Washington, United States of America.; d Health, Law and Development Consultants (HLDC), The Hague, Netherlands.; e Health Systems Development Department, World Health Organization Regional Office for the Eastern Mediterranean, Cairo, Egypt.

## Abstract

Law lies at the centre of successful national strategies for prevention and control of noncommunicable diseases. By law we mean international agreements, national and subnational legislation, regulations and other executive instruments, and decisions of courts and tribunals. However, the vital role of law in global health development is often poorly understood, and eclipsed by other disciplines such as medicine, public health and economics. This paper identifies key areas of intersection between law and noncommunicable diseases, beginning with the role of law as a tool for implementing policies for prevention and control of leading risk factors. We identify actions that the World Health Organization and its partners could take to mobilize the legal workforce, strengthen legal capacity and support effective use of law at the national level. Legal and regulatory actions must move to the centre of national noncommunicable disease action plans. This requires high-level leadership from global and national leaders, enacting evidence-based legislation and building legal capacities.

## Résumé

Le droit est au cœur des stratégies nationales efficaces de lutte contre les maladies non transmissibles. Par droit, nous entendons les accords internationaux, les législations nationales et infranationales, les réglementations et autres instruments exécutifs, et les décisions des cours et des tribunaux. Cependant, le rôle vital du droit dans le développement de la santé à l'échelle mondiale est souvent mal compris, et éclipsé par d'autres disciplines telles que la médecine, la santé publique et l'économie. Cet article définit des domaines d'intersection clés entre le droit et les maladies non transmissibles, en commençant par le rôle du droit en tant qu'outil pour mettre en œuvre des politiques visant à prévenir et maîtriser les principaux facteurs de risque. Nous mettons en évidence des mesures que l'Organisation mondiale de la Santé et ses partenaires pourraient prendre pour mobiliser les professionnels du droit, renforcer les capacités juridiques et soutenir une utilisation efficace du droit au niveau national. Des mesures juridiques et réglementaires doivent être placées au centre des plans d'action nationaux pour la lutte contre les maladies non transmissibles. Cela nécessite un leadership de haut niveau de la part des dirigeants internationaux et nationaux, à travers l'adoption de lois fondées sur des données scientifiques et un renforcement des capacités juridiques.

## Resumen

La ley es la clave del éxito de las estrategias nacionales para la prevención y el control de las enfermedades no contagiosas. Por ley entendemos los acuerdos internacionales, la legislación nacional y subnacional, los reglamentos y otros instrumentos ejecutivos, así como las decisiones de los tribunales y las cortes de justicia. Sin embargo, el papel vital de la ley en el desarrollo de la salud mundial a menudo no se comprende bien y se ve eclipsado por otras disciplinas como la medicina, la salud pública y la economía. Este documento identifica las áreas clave de intersección entre la ley y las enfermedades no contagiosas, empezando por el papel de la ley como herramienta para implementar políticas de prevención y control de los principales factores de riesgo. Se determinan las medidas que la Organización Mundial de la Salud y sus asociados podrían adoptar para movilizar al personal legal, fortalecer la capacidad jurídica y apoyar el uso eficaz de la legislación a nivel nacional. Las acciones legales y reglamentarias deben pasar a ser el centro de los planes de acción nacionales para las enfermedades no contagiosas. Esto requiere un liderazgo de alto nivel por parte de los líderes mundiales y nacionales, para promulgar una legislación basada en pruebas y crear capacidades jurídicas.

## ملخص

يمثل القانون حجر الزاوية بالنسبة للاستراتيجيات الوطنية الناجحة للوقاية من الأمراض غير المعدية ومكافحتها. والمقصود بالقانون هو الاتفاقات الدولية، والتشريعات الوطنية ودون الوطنية، واللوائح وغيرها من الوسائل الإدارية وقرارات المحاكم والهيئات القضائية. ورغم ذلك، فإن الدور الحيوي للقانون في مجال التنمية الصحية العالمية غالباً ما يكون غير مفهوم بشكل جيد، ويتفوق عليه التخصصات الأخرى مثل الطب والصحة العامة وعلم الاقتصاد. يحدد هذا البحث المجالات الرئيسية للتداخل بين القانون والأمراض غير المعدية، بدءا من دور القانون كأداة لتنفيذ سياسات الوقاية والتحكم في عوامل الخطر الرئيسية. نحن نحدد الإجراءات التي يمكن أن تتخذها منظمة الصحة العالمية وشركاؤها لتعبئة القوى العاملة القانونية، وتعزيز الصلاحيات القانونية ودعم الاستخدام الفعال للقانون على المستوى الوطني. يجب أن تكون الإجراءات القانونية والتنظيمية في قلب خطط العمل الوطنية للامراض غير المعدية. ويتطلب ذلك قيادة عالية المستوى من القادة العالميين والوطنيين، وسن تشريعات قائمة على الأدلة، وبناء الصلاحيات القانونية.

## 摘要

法律是成功制订预防和控制非传染性疾病的国家战略核心。我们所说的法律是指国际协议、国家和地方法律、法规和其它执行文书,以及法院和法庭的判决。但是,人们对法治在全球卫生发展中的重要作用知之甚少,且法学的作用往往被医学、公共卫生和经济学等其它学科所替代。本文确定了法律与非传染性疾病交叉的关键领域,以法治工具作为实施预防和控制主要风险因素政策的开端。我们明确世界卫生组织及其合作伙伴可以采取行动来动员合法劳动力,加强法律能力并支持法律在国家层面上的有效利用。法律和法规行为必须转向国家非传染性疾病行动计划的中心。这需要在国际和国家层面上实施基于实证的立法和建设法律能力的高层领导。

## Резюме

Закон лежит в основе успешных национальных стратегий по профилактике и борьбе с неинфекционными заболеваниями. Под законом подразумеваются международные соглашения, национальное и субнациональное законодательство, нормативные акты и другие постановления исполнительных органов, а также решения судов и трибуналов. Однако исключительно важная роль закона в области развития глобального здравоохранения часто плохо осознается и перекрывается такими дисциплинами, как медицина, общественное здравоохранение и экономика. Настоящий документ определяет основные области пересечения между законом и лечением неинфекционных заболеваний, начиная с роли закона как инструмента для реализации стратегий по профилактике и борьбе с ведущими факторами риска. Авторы определяют меры, которые могут быть предприняты Всемирной организацией здравоохранения и ее партнерами по мобилизации юридического персонала, укреплению правоспособности и поддержке эффективного использования закона на национальном уровне. Правовые и нормативные меры должны стать основой для разработки национальных планов действий по борьбе с неинфекционными заболеваниями. Для этого необходимо заручиться поддержкой мировых и национальных лидеров, обеспечить принятие научно обоснованных законов и развитие правового потенциала.

## Introduction

Noncommunicable diseases, including cardiovascular disease, cancer, respiratory diseases and diabetes, cause an estimated 41 million deaths each year.^1^ Fifteen million of these deaths occur in people aged 30–69 years,^1^ at a time of life when people are working and more likely to have dependants. Over 12 million (85%) of these premature deaths occur in low- and middle-income countries, where health systems may be fragile and access to treatments suboptimal.^1^ So far, progress towards global goals (Box 1) and political commitments on noncommunicable diseases has been disappointing.^4^ Unless urgent action is taken, the burden of mortality and disability from noncommunicable diseases will increase substantially, driven by population growth, longer life-expectancies and the global diffusion of risk factors such as tobacco use, harmful alcohol use, obesity, poor diet and sedentary lifestyles.^5^

Box 1 **Global targets for reductions in noncommunicable disease risk factors**

**Global Monitoring Framework on noncommunicable diseases (World Health Organization)^2^**

Overall target:

- by 2025, a 25% relative reduction in mortality from cardiovascular disease, cancer, diabetes and chronic respiratory diseases in persons aged 30–70 years.

Eight supporting targets:

- 10% relative reduction in harmful use of alcohol;
- 10% relative reduction in prevalence of physical inactivity;
- 30% relative reduction in mean average population salt intake;
- 30% relative reduction in prevalence of tobacco use (persons older than 15 years);
- 25% relative reduction in raised blood pressure;
- 0% increase in diabetes and obesity;
- 50% coverage for drug therapy and counselling for those at risk for cardiovascular disease;
- 80% coverage of affordable technologies and essential medicines for treating noncommunicable diseases in both public and private facilities.

**Sustainable development goal 3 (United Nations)^3^**

Target 3.4:

- by 2030, reduce by one third premature mortality from noncommunicable diseases through prevention and treatment and promote mental health and wellbeing.

The effective use of law and regulation lies at the heart of successful national noncommunicable disease action plans.^6^ Law includes international agreements, national and subnational legislation, subsidiary legislation (also known as regulations) and other executive instruments, and decisions of courts and tribunals. As a broader concept, regulation includes legislation, fiscal policies (such as taxes and subsidies) and other legally binding standards. Recognizing the power of law to improve the public’s health, the World Health Organization (WHO) offers technical assistance to governments on appropriate legal strategies (Box 2). Constraints and challenges in using law effectively at the national level include the lack of personnel with legal training or expertise, lack of resources for enforcement, and the influence of vested commercial interests in drafting, implementing and enforcing laws. Despite these challenges, law remains an important tool for taking action to reduce the burden of these diseases. Encouragingly, heads of state and governments have committed to promoting and implementing “policy, legislative, and regulatory measures, including fiscal measures”^10^ to address noncommunicable disease risk factors and to developing legal expertise to integrate “public health-related legal issues into noncommunicable disease country support.”^11^

Box 2 **Scope of technical work on law and noncommunicable diseases by the World Health Organization**

The World Health Organization’s (WHO) work at the intersection of law and noncommunicable diseases includes:

- supporting governments to develop laws and regulations on health matters through technical assistance, training and provision of technical resources;
- comparative analysis of laws in different jurisdictions for WHO publications, including biannual reports on the global tobacco burden;
- analysing litigation and industry opposition to policies and laws, and integrating lessons learnt into technical assistance and resources;
- gathering evidence in support of effective public health laws and policies in Member States;
- providing assistance to Member States in litigation matters;
- intervening in legal disputes; for example, through the amicus briefs filed in disputes over tobacco control laws^7^^–^^9^; and
- engaging with other inter-governmental organizations on legal and normative issues.

This paper identifies some important areas of intersection between law and prevention and control of noncommunicable diseases, arguing that law lies at the centre of effective action. We suggest actions that WHO and other health development partners could take to strengthen national legal capacities and accelerate implementation of legal and regulatory strategies.

## Law and noncommunicable diseases

### Implementing preventive policies

In 2017, the World Health Assembly endorsed an updated set of policy options and cost–effective interventions for reducing the burden of noncommunicable diseases, including minimizing the major risk factors (consumption of tobacco, alcohol, unhealthy foods and drinks high in sugar).^12^ Many of these recommended policies are legal interventions, requiring legislation or executive actions for effective implementation (Box 3). Sales of tobacco, alcohol, processed foods and sugar-sweetened drinks have expanded rapidly in low- and middle-income countries because of trade and investment liberalization, leading to greater foreign direct investment, imports and advertising.^14^^,^^15^ Implementing WHO’s evidence-based, highly cost-effective policy interventions (called best-buys) should be a priority for governments, but requires political commitment, funding and strong legal capacity.

Box 3 **Legally oriented best-buys and policy interventions for noncommunicable disease prevention and control**

Highly cost–effective policy interventions (best-buys) recommended by the World Health Organization include:

- increasing excise taxes and prices of tobacco products and alcoholic beverages;
- eliminating peoples’ exposure to second-hand tobacco smoke in all indoor workplaces, public places and public transport;
- comprehensive bans on tobacco advertising, promotion and sponsorship;
- implementing plain tobacco packaging and/or graphic health warnings on all tobacco packages;
- comprehensive bans or restrictions on alcohol advertising across multiple forms of media;
- enforcing restrictions on the physical availability of alcohol via reduced hours of sale in retail outlets;
- mandatory product reformulation and front-of-pack labelling to help people to reduce salt intake.

Other cost–effective policy interventions include:

- implementing and enforcing drink-driving laws and blood alcohol concentration limits via sobriety checkpoints;
- eliminating industrial trans-fats through legislation banning their use in food manufacturing;
- taxation of sugar-sweetened beverages at a rate high enough to encourage people to reduce sugar consumption.

Other recommended, legally-oriented policy options include:

- measures to minimize illicit trade in tobacco products;
- bans on cross-border tobacco advertising;
- setting minimum alcohol prices;
- enforcing a minimum age for purchase of alcoholic beverages;
- reducing the density of retail alcohol outlets;
- restricting or banning promotions of alcoholic beverages targeting young people;
- requiring labels for alcoholic beverages to include information about the harm caused by alcohol;
- implementing subsidies to increase people’s intake of fruits and vegetables;
- replacing trans-fats and saturated fats with unsaturated fats through mandatory reformulation, product labelling, fiscal or agricultural policies;
- implementing nutrition labelling to encourage people to reduce consumption of energy, sugars, sodium and fats;
- improving urban environments to ensure ease of walking, connectivity and access to public transport to encourage people to engage in more physical activity.

Source: World Health Organization.^13^

The force of law is needed to implement effective policies for prevention and control of noncommunicable disease risks because voluntary implementation will rarely be in the commercial interests of food, alcohol and tobacco companies. There are similarities in the ways these industries seek to influence not only legislative outcomes, but also the perceptions of politicians and the public, the framing of issues for debate and the generation of favourable evidence.^16^^,^^17^ A growing literature illustrates the ways in which these industries, their allies and proxies, lobby governments, donate to political campaigns and undermine scientific evidence, seeking to shift the public focus from healthy public policies towards personal responsibility and fears of paternalism.^16^^,^^17^ Industry may also seek to pre-empt enforceable standards by implementing weaker, self-regulatory codes or may lobby to increase industry influence through partnerships and co-regulatory approaches.^18^ When regulating and dealing with these industries, governments and international institutions should implement rigorous conflict-of-interest policies to avoid inappropriate forms of influence.^16^

Apart from seeking to reduce noncommunicable disease risk factors, law is an important tool for establishing institutional and governance mechanisms to support public health functions. For example, Samoa’s Health Promotion Act, enacted in 2013, established the Samoa Health Promotion Foundation, giving it a legislative mandate to engage in health promotion, fund research and advise the health minister.^19^ Through executive action, governments can also establish a national coordination mechanism to implement a whole-of-government approach to policy implementation that ensures policy coherence and mutual accountability of those ministries that have a bearing on noncommunicable disease risks. An example is Mexico’s National Council for the Prevention and Control of Chronic Noncommunicable Diseases, which brings together the heads of national executive agencies to coordinate cross-sectoral actions and policies.^20^ The WHO Independent High-level Commission on noncommunicable diseases has emphasized that action must start at the top, with the president or prime minister of a country leading a multisectoral national response.^4^

### Constitutional rights

The constitution of many countries guarantees health-related rights to their population.^21^ Constitutional rights not only limit parliamentary powers, but may permit individuals or groups to claim remedies for interference with their rights. In such countries, health-related rights may provide one avenue for challenging actions (and omissions) by governments and corporations that are harmful to health, as seen in India and Uganda (Box 4).^22^^–^^24^

Box 4 **Examples of constitutional rights litigation affecting noncommunicable disease risk factors**

Constitutional rights may provide remedies for groups harmed by government or corporate actions or omissions:

**India**

In India, individuals may petition the Supreme Court to enforce fundamental rights and liberties in the Indian Constitution. The Supreme Court has ruled that the exposure of non-smokers to tobacco smoke violates the constitutional right to life and personal liberty.^22^ The Court issued an order, subsequently implemented through national legislation, required federal and state governments to implement smoking bans in several public settings. The Supreme Court has also held that the right to life in the Constitution encompasses a right to live with human dignity, which encompasses the right to food. In a series of orders, the Court has expanded coverage and legal entitlements under food assistance programmes.^23^

**Uganda**

The High Court of Uganda has ruled that the emission of tobacco smoke, dust and smell from a tobacco factory in a residential area violates the right to a healthy and clean environment, as protected in the Ugandan Constitution.^24^ Finding that the National Environment Management Authority had failed in its responsibilities, the court ordered the relocation of the factory.

**Colombia**

A Colombian civil society organization, Educar Consumidores, successfully challenged an order given by a regulatory authority, the Superintendency of Industry and Commerce, directing Educar Consumidores to cease transmission of a television and radio campaign that pointed to the quantities of sugar in sugary drinks and their harmful effects. Educar Consumidores and representatives of another civil society organisation successfully petitioned for review by the Colombian Constitutional Court, which held that the ban violated consumers’ constitutional right to receive information – and to inform others – about the health risks of sugary drinks.^25^

On the other hand, constitutional rights may conflict with public health policies on noncommunicable diseases:

**United States of America**

The city of San Francisco passed a local ordinance requiring billboards advertising soda drinks to display a warning that added sugars contribute to obesity, diabetes and tooth decay. In response, the American Beverage Association obtained an injunction on the basis that a mandatory warning infringed their First Amendment rights to free speech.^26^ An appeals court has agreed to re-hear this case.

On the other hand, national constitutions typically protect a range of non-health-related rights that may conflict with public health policies. In some countries, the right to freedom of expression protects commercial speech, which may undermine efforts to restrict the marketing of tobacco, alcohol, unhealthy food and drinks, and breastmilk substitutes. Civil society organizations have also relied successfully on the right to freedom of expression to resist efforts to supress information about the health effects of sugary drinks, as illustrated by an example from Colombia (Box 4).^25^

### International human rights

International law can also influence national policies on noncommunicable disease prevention and control. For example, parties to the WHO Framework Convention on Tobacco Control have assumed an obligation under international law to implement policies on reduction in demand and supply of tobacco products.^27^ Most countries have also ratified at least one international agreement recognizing the right to health or other health-related rights. Such agreements include the WHO Constitution, various United Nations (UN) conventions (e.g. the International Covenant on Economic, Social and Cultural Rights; the Convention on the Rights of the Child; the Convention on the Elimination of All Forms of Discrimination Against Women) and regional agreements (e.g. the African Charter on Human and People’s Rights).

Unlike the WHO Framework Convention on Tobacco Control, health-related provisions in human rights treaties are often expressed in general terms and were not framed with noncommunicable disease risk factors in mind.^28^ Nevertheless, the Committee on Economic, Social and Cultural Rights, the treaty-monitoring body for the International Covenant on Economic, Social and Cultural Rights, has identified core obligations arising under the right to health that are directly relevant to noncommunicable diseases. These include the obligation to ensure access to health services without discrimination; nutritionally adequate food; safe and potable water; and essential medicines.^29^ Under the Covenant, UN Member States also have an obligation to respect, protect and fulfil the right to health. The obligation to protect requires countries to prevent third parties, including corporations, from violating this right. Member States must also remedy regulatory failures, such as “failure to discourage production, marketing and consumption of tobacco, narcotics and other harmful substances.”^29^ The obligation to fulfil the right to health addresses the problem of inaction by Member States, requiring them to adopt “legislative, administrative, budgetary, judicial, promotional and other measures” towards full realization of the right.^29^

Each country’s compliance with its human rights obligations is reviewed through a process known as Universal Periodic Review, overseen by the UN Human Rights Council. Universal Periodic Review provides an impetus for governments to strengthen health-related rights,^30^ and may assist governments defending rights-based claims made by corporations, such as freedom of expression or property rights. During this process, human rights treaty bodies may draw attention to priority risk factors, urging Member States to implement effective policies on noncommunicable diseases. For example, concerned about increasing food insecurity (including its link with obesity) and low levels of breastfeeding, the Committee urged the United Kingdom of Great Britain and Northern Ireland to implement national policies on breastfeeding in accordance with World Health Assembly resolutions and the International Code of Marketing of Breast-milk Substitutes; increase taxes on unhealthy foods and sugar-sweetened drinks; and “consider adopting strict regulations on the marketing of such products, while ensuring improved access to healthy diets.”^31^ Similarly, in 2014, the Committee expressed concern about tobacco addiction in Indonesia, recommending indoor smoking bans in public buildings and workplaces, and a ban on tobacco advertising and sponsorship.^32^ In 2007, the Committee on the Rights of the Child recommended that Chile “take necessary measures to reduce and prevent the incidence of obesity among children.”^33^ This report could strengthen Chile’s position in counteracting litigation initiated by food producers against the country’s restrictions on marketing to children.

International human rights law and practice is a neglected resource for governments seeking to reduce the burden of noncommunicable diseases. Analysis of specific health rights within international human rights agreements provides important guidance in many areas, such as tobacco control and marketing of foods and beverages.^34^ International treaty bodies could make greater use of the WHO Framework Convention on Tobacco Control^27^ and authoritative sources of guidance on risk factors, including the Global Action Plan on Prevention and Control of noncommunicable diseases,^2^ when evaluating Member States’ compliance with their obligations under human rights treaties.

### Trade and investment agreements

International trade law is another important area of law that impacts on risk factors for noncommunicable diseases. International trade and investment agreements can affect noncommunicable disease risk factors in complex ways; for example, by reducing prices, increasing competition, and facilitating international trade and investment in harmful products, such as tobacco and alcohol.^35^

In legal terms, trade and investment agreements discipline how states can regulate. For example, World Trade Organization (WTO) law establishes core principles of trade law, including prohibiting discriminatory regulation, requiring that regulation be not more trade restrictive than necessary to protect health, and requiring minimum standards of protection for intellectual property rights. The WTO panels have applied these principles in adjudicating legal disputes concerning tobacco control. For example, a WTO panel held that United States legislation banning flavoured tobacco, but exempting menthol-flavoured products discriminated in favour of domestic products.^36^ More recently, a WTO panel upheld Australia’s tobacco plain packaging laws as not more trade restrictive than necessary and not an unjustifiable encumbrance on the use of trademarks.^7^ WTO committees have also discussed legal measures to reduce harmful alcohol use^37^ and improve healthy diets,^38^ although such measures have not resulted in WTO disputes.

Regional trade agreements are increasingly important. Customs unions, such as the European Union, can lead to harmonization of laws, such as through the 2014 European Tobacco Products Directive.^39^ However, rules concerning free movement of goods, state aid and communications can also be used to challenge fiscal and regulatory measures. Examples include the (recent unsuccessful) challenge to the introduction of minimum unit pricing on alcoholic beverages in Scotland,^40^ limitations on Sweden’s ability to restrict alcohol marketing originating in the United Kingdom,^41^ and the scrapping of a Finnish confectionary tax because of rules concerning state aid.^42^

Investment treaties, whether bilateral or as investment chapters in trade agreements, can also impact on legislation concerning noncommunicable diseases. The tobacco company Philip Morris International recently challenged tobacco control laws in Australia and Uruguay. These claims, which were unsuccessful but expensive to defend, concerned plain tobacco packaging, health warnings and other labelling requirements implemented in response to the WHO Framework Convention on Tobacco Control.^8^^,^^9^

### Litigation and complaints mechanisms

Challenges to policies for prevention and control of noncommunicable diseases are frequently resolved in national courts and tribunals, as illustrated again by the alcohol industry’s 5-year attempt to overturn Scotland’s legislation on minimum alcohol pricing.^43^ The tobacco, food and alcohol industries are increasingly suing governments, relying on constitutional and human rights guarantees, although with mixed success.^44^

On the other hand, litigation and use of other complaints mechanisms can be used to hold industry to account for harm caused by their products, to improve access to medicines and to vindicate other health-related rights.^6^ Tobacco litigation in the United States has had a major impact. A Department of Justice lawsuit found that for half a century the tobacco industry engaged in a pattern of fraudulent conduct to deceive the American public about the effects of cigarettes on health.^45^ Complaints made under consumer protection laws are another underused tool. For example, an Australian court imposed a fine of more than 2 million Australian dollars against the Heinz food company for engaging in false and misleading conduct by advertising that a chewy fruit snack, containing about two-thirds sugar, was beneficial to the health of children aged 1–3 years.^46^

## Effective use of law

Legislation and executive actions are essential for noncommunicable disease prevention and control, while many of the challenges to effective national plans, such as defending litigation initiated by industry, also call for technical legal expertise. However, governments face major obstacles to using law effectively, including lack of trained personnel, lobbying by powerful industry groups and uncertainty about the extent of their country’s obligations under trade and investment agreements.^16^ Nevertheless, as Box 5 illustrates, governments can overcome these challenges and pass innovative laws for prevention and control of noncommunicable disease risk factors.

Box 5 **Examples of innovation in legal and regulatory approaches to prevention and control of noncommunicable diseases**

**Chile**

In 2015, the Chilean Ministry of Health published regulations on the nutritional composition of food products, implementing an earlier law passed in 2012.^47^^,^^48^ These laws require packaged food that exceeds limits set (per 100 g or per 100 mL) for energy, sodium, sugar or saturated fat, respectively, to be prominently labelled as “high in” each of these nutrients. The same law prohibits food advertising that is directed at children younger than 14 years where the food exceeds the limits for energy, sodium, sugar or saturated fat. Finally, foods that exceed the cut-off points for over-consumed nutrients cannot be sold in pre-school, primary or secondary school.

**Mexico**

Mexico’s 1 peso per litre tax on sugar-sweetened drinks, implemented in 2014, resulted in a seasonally adjusted reduction in consumption of taxed beverages of 5.5% in 2014, and 9.7% in 2015, compared with estimates based on trends in consumption before the implementation of the tax.^49^

**South Africa**

In 2013, South Africa introduced regulations under its Foodstuffs, Cosmetics and Disinfectants Act to impose maximum limits for sodium across 13 categories of food including bread, breakfast cereal and porridge, processed meat, savoury snacks and potato chips.^50^ These limits took effect in June 2016, with lower limits to be phased in from 30 June 2019. One study estimated these regulations could avoid 5600 cardiovascular disease deaths per year.^51^

**United Kingdom of Great Britain and Northern Ireland**

Effective from April 2018, the soft drinks levy introduced in the United Kingdom has two tax levels that apply according to the sugar content: 18 pence for drinks with > 5 g sugar per 100 mL, and 24 pence for drinks with > 8 g sugar per 100 mL.^52^ The levy incentivized reformulation of products by soft-drink manufacturers even before the tax took effect, reducing expected revenues from pounds sterling (£) 520 to £240 million in the first year of the law.

**United States of America**

Six States (California, Hawaii, Maine, Massachusetts, New Jersey, Oregon) and more than 350 cities and counties, including New York city, have raised the minimum purchasing age for tobacco from 18 to 21 years.^53^ In California and New York city, a minimum age of 21 years is required to purchase both cigarettes and electronic cigarettes.^54^^,^^55^

Public health advocates have called on the World Health Assembly to make bold use of its legal powers, through a convention on alcoholic beverages^56^ or obesity^57^ and unhealthy diets. A global coordinating agency for noncommunicable diseases has also been proposed, to encourage public and private financing, aligned with the sustainable development goals.^58^ However, even in the absence of these major reforms, we see opportunities for governments and their development partners, including WHO, to strengthen national noncommunicable disease prevention and control.

### Sharing good practices

The WHO Independent High-level Commission on noncommunicable diseases encouraged governments to use their “full legal and fiscal powers to achieve public health goals.”^4^ This includes regulating harmful products and practices, and strengthening the institutions, functions and official roles that are needed to ensure compliance with legal standards.^6^ Effective use of law requires budgetary resources for enforcement, including training. Distinct from its law-making role, WHO’s power to develop soft (non-binding) normative standards, together with its capacity to convene and disseminate expert knowledge, remain powerful tools. WHO is already developing technical resources to support regulatory actions by governments; for example, implementing plain packaging of tobacco products^59^ and the International Code of Marketing of Breast-milk Substitutes.^60^ WHO, with its development partners, should invest further to increase technical resources in law and regulation.

WHO could also facilitate the diffusion of policy across Member States by creating opportunities for governments that are leading the way to share their practical experiences of drafting, implementing and enforcing laws and fiscal policies.^10^ Since legal systems vary widely, such exchanges among officials and stakeholders in different countries are likely to be more productive than exhaustively cataloguing health laws or promoting model legislation. Recent experience with plain packaging of tobacco products shows that technical activities such as training workshops, online platforms linking people who work in a common field and comparative analysis of laws and litigation can also facilitate policy diffusion.

### Mobilizing the legal workforce

Building legal capacity means mobilizing a workforce with the technical skills to navigate legal issues arising in key areas of noncommunicable disease prevention and control.^61^ Such efforts could help to translate evidence and WHO guidance into country-level action. Creating a platform for lawyers, legislators, educators and public health experts to interact may not only improve the exchange of advice and information across governments, civil-society organizations and academia, but also provide mentors for young leaders working on reform of laws on noncommunicable diseases in low-resource countries.

Leadership requires greater investment in legal capacity within WHO and other agencies working in health development, and careful assembling of a legal workforce. Donor funding for a dedicated public health law programme within WHO to support countries in implementing best-buy policy interventions could have a substantial impact.^62^ Within available resources, we see two key areas where global leadership could strengthen legal capacities.

First, WHO could facilitate professional exchanges and mutual support among those who use legal knowledge in key practice areas. Fostering such a transnational network could be achieved online, with support for legal practitioners working in key thematic areas, countries and regions, and in different languages. With appropriate support, these communities of practice will grow and evolve, seeking out strategic opportunities, developing novel legal arguments, providing expert evidence in litigation (through filing of amicus briefs), documenting case studies and developing technical resources in areas of need.

Second, knowledge provides the foundation for action. In the context of tobacco control, for example, databases of case law and legislation facilitate comparative analysis and assist lawyers and health professionals to anticipate tobacco industry strategies.^63^ No comparable resource exists for taking action on unhealthy diets or harmful use of alcohol. Consequently, industry legal arguments are neither systematically tracked nor easily anticipated.

### Building on local innovations

Although prevention and control of noncommunicable diseases ideally require a national response, state, city and local governments can be powerful innovators (Table 1). Unlike national governments, smaller, defined localities often have more homogeneous constituencies, smaller and more efficient administrations and less time-consuming legislative processes. In response to political activism, social mobilization and specific social, economic and demographic factors, local and city administrations can become laboratories for innovation, trialling new legal approaches. The impact of these local innovations can be evaluated, disseminated and implemented both horizontally (to other localities) and vertically (at state and national levels).^64^

**Table 1:** Examples of local innovations in prevention and control of noncommunicable diseases in the United States of America

Policy innovation and example	Description	Reference
**Information disclosure**		
Calorie labelling rules	Restaurant chains and food retailers in New York city must disclose calorie counts on menu boards for standard menu items	New York City Health Code §81.50 (2017)
Soda warning rule	Billboards advertising sugar-sweetened drinks in San Francisco city must contain a health warning about the impact of added sugars on obesity, diabetes and tooth decay	San Francisco Health Code art. 42 §4203(a) (2015)
Haemoglobin A1C registry	New York city’s health code makes glycated haemoglobin (a measure of blood sugar control) a reportable condition by pathology laboratories. The registry helps to identify patients with poorly controlled diabetes or who need follow-up care	New York City Health Code §13.07 (2006)
**Marketing restrictions**		
School advertising law	Maine was the first state to prohibit brand-specific advertising of food or beverages in school buildings or on school grounds	Title 20-A Maine Rev. Stat. Ann §6662 (2007)
Healthy food incentives ordinance	Fast-food restaurants in San Francisco city are prohibited from providing free toys in children’s meals	San Francisco Health Code art. 8 §§471.1 to 471.9 (2011)
**Taxation**		
Sugar-sweetened beverage tax	The city of Berkeley was the first jurisdiction in the country to impose an excise tax of 1 cent per ounce on sugar-sweetened drinks	Berkeley Municipal Code Chapter 7.72 (2014)
Sugar-sweetened beverage tax	Philadelphia was the first major city to levy a tax of 1.5 cents per ounce on sugar-sweetened drinks and to earmark tax revenue for improvements to parks, libraries and recreation centres	Philadelphia Code §§19–4101 to 4108 (2016)
**Built environment: zoning**		
Urban agriculture incentives	California State’s Urban Agriculture Incentive Zones Act reduces property taxes for landowners who enter a contract to permit small-scale agriculture or animal husbandry for at least 5 years on vacant lands	Cal Govt Code §51042 (2017
Ordinance to control prevalence of fast-food outlets	The city of Los Angeles limits new fast-food restaurants in areas with an over-concentration of fast-food outlets	L.A. Cal. Ordinance 180103 (2008)

§: section; §§: sections.

Local governments have been leaders in many areas of noncommunicable disease policy. In the United States, local jurisdictions were the first to impose higher taxes, marketing restrictions and bans on smoking in public places, among other interventions.^65^ Some cities and towns have adopted a range of additional strategies, such as banning trans-fatty acids from the food supply, raising the minimum purchasing age for tobacco products and imposing health-based taxes on sugar-sweetened drinks.^66^

### Coordinating regional action

Regionally coordinated action can accelerate the implementation of legislation, particularly by smaller countries. The best opportunities exist in regions that have a strong history of cooperative action. WHO’s regional offices and other regional political groupings (e.g. the Pacific Community, the Caribbean Community, the African Union, the Association of Southeast Asian Nations or the Organization of American States) could lead, coordinate and support national noncommunicable disease policies. Deliberate, planned, regional action could benefit small, remote and vulnerable populations whose governments may otherwise engage in prevention and control in a piecemeal fashion and at a slower rate. Regional action allows more efficient use of legal resources, although careful groundwork is needed to create a shared vision and agreed principles for action. Box 6 summarizes an example from WHO’s Regional Office for the Eastern Mediterranean.

Box 6 **Example of effective and regionally relevant legal interventions for control of noncommunicable diseases by WHO**

The World Health Organization Regional Office for the Eastern Mediterranean (WHO-EMRO), partnered with the O’Neill Institute for National and Global Health Law at Georgetown University, Washington, United Sates of America, to spur legal reform for noncommunicable disease prevention and control. The project identified affordable, feasible and cost–effective legislative and regulatory interventions that were suitable for implementation in the Eastern Mediterranean Region.

The comprehensive dashboard of legal interventions proposed that WHO-EMRO Member States prioritize interventions in three key domains: (i) noncommunicable disease governance mechanisms requiring multisectoral collaboration, accountability and transparency; (ii) tobacco control laws in compliance with the WHO Framework Convention on Tobacco Control;^27^ and (iii) laws to promote healthier diets, such as reducing consumption of sodium and sugar.^67^

WHO-EMRO committed to support Member States to enact and enforce population health improvements through these priority interventions. The project includes implementation guidance tools as well as capacity-building initiatives led by multidisciplinary teams of legislators and public health experts.^68^ The EMRO–O’Neill Institute partners published a detailed description of the evidence-based legal policies to reduce noncommunicable disease prevalence in the Region.^69^

## Conclusion

Legislative and regulatory actions lie at the heart of successful national and local strategies for noncommunicable disease prevention and control. However, the role of law as a public policy tool, translating scientific evidence and normative guidance into action, receives inadequate attention amid the dominance of other disciplines in global health development. This needs to change. Rapid progress in tobacco control has not been accidental, but reflects agreement about the critical importance of law to tobacco control – leading, in turn to adoption of strong, legally-binding standards at international and national levels. The rapid progress also reflects global investment in capacity-building, technical assistance to WHO Member States, and expansion of legal resources, assisted by organizations such as Bloomberg Philanthropies and the Bill & Melinda Gates Foundation. These factors, which have accelerated progress in tobacco control, are not yet present for other noncommunicable disease risk factors such as unhealthy diets and harmful use of alcohol. Legal and regulatory action must move to the centre of national noncommunicable disease action plans. This requires high-level leadership at global and national levels, developing evidence-based legislation through transparent processes, enforcing it, evaluating its effectiveness and building legal capacities.
